# Transcriptome Profiling of *Nasonia vitripennis* Testis Reveals Novel Transcripts Expressed from the Selfish B Chromosome, Paternal Sex Ratio

**DOI:** 10.1534/g3.113.007583

**Published:** 2013-09-01

**Authors:** Omar S. Akbari, Igor Antoshechkin, Bruce A. Hay, Patrick M. Ferree

**Affiliations:** * Division of Biology, MC156-29, California Institute of Technology, Pasadena, California 91125; † W. M. Keck Science Department, Claremont McKenna, Pitzer, and Scripps Colleges, Claremont, California 91711

## Abstract

A widespread phenomenon in nature is sex ratio distortion of arthropod populations caused by microbial and genetic parasites. Currently little is known about how these agents alter host developmental processes to favor one sex or the other. The paternal sex ratio (PSR) chromosome is a nonessential, paternally transmitted centric fragment that segregates in natural populations of the jewel wasp, *Nasonia vitripennis*. To persist, PSR is thought to modify the hereditary material of the developing sperm, with the result that all nuclear DNA other than the PSR chromosome is destroyed shortly after fertilization. This results in the conversion of a fertilized embryo—normally a female—into a male, thereby insuring transmission of the “selfish” PSR chromosome, and simultaneously leading to wasp populations that are male-biased. To begin to understand this system at the mechanistic level, we carried out transcriptional profiling of testis from WT and PSR-carrying males. We identified a number of transcripts that are differentially expressed between these conditions. We also discovered nine transcripts that are uniquely expressed from the PSR chromosome. Four of these PSR-specific transcripts encode putative proteins, whereas the others have very short open reading frames and no homology to known proteins, suggesting that they are long noncoding RNAs. We propose several different models for how these transcripts could facilitate PSR-dependent effects. Our analyses also revealed 15.71 MB of novel transcribed regions in the *N. vitripennis* genome, thus increasing the current annotation of total transcribed regions by 53.4%. Finally, we detected expression of multiple meiosis-related genes in the wasp testis, despite the lack of conventional meiosis in the male sex.

During the past several years, the jewel wasp, *Nasonia vitripennis*, has gained attention as a rising insect model for genomic and developmental genetic studies. As a member of the order Hymenoptera, which includes all wasps, bees, and ants, *N. vitripennis*, a wasp that is distributed worldwide, is a close sibling of three other North American wasp species within the *Nasonia* genus (Darling and Werren 1990). All of these wasps are gregarious parasitoids, laying clutches of multiple progeny within the pupae of *Sarcophaga* blowflies (Darling and Werren 1990). Because of their close relatedness, individuals of each *Nasonia* species can be interbred to produce viable F1 hybrid progeny with trait variations that are easily quantified and mapped to gene-level resolution (Gadau *et al.* 1999; Pultz and Leaf 2003; Werren and Loehlin 2009). Due in large part to the completion of the *N. vitripennis* genome (Werren *et al.* 2010b), which has facilitated a growing number of genetic and genomic tools, this species has become a preferred system for investigating the genetic basis of several hymenopteran-related traits. These include venom production, sex determination, and evolutionary development of characteristics such as wing length and axis patterning (de Graaf *et al.* 2010; Loehlin *et al.* 2010; Verhulst *et al.* 2010).

One of the most distinguishing hymenopteran characteristics is haplodiploid reproduction, which likely makes *N. vitripennis* and other members vulnerable to manipulation by microbial and genetic parasites. In haplodiploidy, females develop as diploids from fertilized eggs whereas males arise as haploids from unfertilized eggs. Meiosis in the male sex of hymenopterans occurs as a modified form of mitosis—likely, in part, because of a lack of homolog pairing partners required for bivalent formation at meiosis I (Whiting 1968). A compelling question, therefore, is whether the meiotic genes that are active in females also function in the male germ line, and if so, in what ways. Because the earliest known signal for sex is an interaction between maternal and zygotic factors that are ultimately determined by the number of chromosome sets present in the egg (Verhulst *et al.* 2010), manipulation of chromosomes by parasites during early development can directly influence sex of the individual and have profound effects on population sex ratios. For example, the endocellular bacterium, *Arsenophonus nasoniae*, a natural symbiont of *N. vitripennis*, manipulates key aspects of the mitotic machinery required for haploid development from unfertilized eggs to kill male progeny (Ferree *et al.* 2008). This, in turn, biases host sex ratios toward female, an effect that is thought to benefit the bacteria because they are transmitted solely from infected mother to offspring through the egg cytoplasm, and killing male individuals may provide more resources for the transmitting (female) sex through reduced competition between siblings (Hurst and Jiggins 2000).

Sex ratios of hymenopteran insects also can be influenced by selfish genetic elements including supernumerary B chromosomes. The *N. vitripennis* genome normally contains five chromosomes that are similar in size. However, some individuals also contain a supernumerary (B) chromosome known as paternal sex ratio (PSR). Structurally, PSR is a small, centric fragment that may have arisen from a normal chromosome through an ancient genome fragmentation event (Werren and Stouthamer 2003). Unlike sex ratio-distorting bacteria, which are primarily maternally transmitted, PSR is transmitted paternally through the sperm (Nur *et al.* 1988). Previous studies have demonstrated that PSR is nonessential for the fitness of *N. vitripennis* (Reed and Werren 1995; Werren *et al.* 1987). To persist, PSR modifies the paternal chromatin in some unknown way so that it is completely destroyed during the first mitotic division of the fertilized embryo. Specifically, during prophase the paternal chromatin fails to resolve into distinct chromosomes and becomes abnormally hypercondensed while the maternal chromosomes resolve normally (Reed and Werren 1995; Werren *et al.* 1987). In insects, the two parental chromosome sets remain separated by a layer of incompletely degraded nuclear envelope during the first division (Callaini and Riparbelli 1996). This feature allows the maternal chromosomes to segregate normally into daughter nuclei, whereas the modified paternal chromatin mass remains at the metaphase plate and is lost during subsequent cleavage divisions (Reed and Werren 1995). Due to the haplodiploid nature of *N. vitripennis*, these fertilized embryos, which should become diploid females, are converted into PSR-transmitting haploid males. PSR somehow spares itself this hypercondensed fate despite its close association with the sperm nuclear material, and it joins the maternal set during the first mitosis to be transmitted (Swim *et al.* 2012). Because of this severe effect on the paternal half of the genome, PSR is considered to be the most extreme genetic element known in nature (Werren and Stouthamer 2003). Additionally, the effect of PSR is a striking example of intragenomic conflict, a condition in which an individual part of the genome achieves enhanced transmission at the expense of the genome as a whole.

A fundamental question is how PSR modifies the paternal chromatin at the molecular level. Several important clues were provided by a recent study, which showed that in the presence of PSR, histone H3 of the paternal set abnormally retains its phosphorylated state after exit from the first embryonic mitosis and persists in this phosphorylated state throughout multiple cell cycles before the chromatin becomes lost (Swim *et al.* 2012). Additionally, the Condensin complex, which functions in eukaryotes to resolve chromatin into distinct chromosomes, becomes overly concentrated on the paternal chromatin and persists in a manner that mirrors the abnormal paternal histone H3 pattern (Swim *et al.* 2012). However, the paternal set appears to replicate properly and does not become abnormally phosphorylated before entry into the first mitosis (Swim *et al.* 2012). These latter observations are consistent with the idea that defective PH3 and Condensin complex retention occur secondarily due to some other chromatin modification that is established at an earlier developmental time (Swim *et al.* 2012).

The close association of PSR with the sperm nuclear material has led to speculation that the initial PSR-induced modification occurs at some point during spermatogenesis (Werren and Stouthamer 2003). Unlike Drosophila males, which produce sperm continually as adults, Nasonia males produce the majority of their sperm during the pupal stage (Whiting 1968). Division of stem cells at the anterior region of the testis produces spermatogonia, which divide mitotically with incomplete cytokinesis to form cysts of premeiotic spermatocytes. The cells within all of these cysts progress through meiosis synchronously to produce haploid spermatids (Swim *et al.* 2012). These cells and their nuclei become highly elongated before becoming individualized into mature sperm. During spermatid nuclear elongation in most animals, the paternal DNA is stripped of its conventional histones, and it is repackaged with special histone-like proteins known as protamines. In some organisms, a small fraction of histones H3 and H4 remain within the sperm chromatin (Dorus *et al.* 2006; Gatewood *et al.* 1987; Govin *et al.* 2007). These histones can retain unique posttranscriptional modifications, such as methylation or acetylation of specific Lysine residues, which are established during earlier stages of spermatogenesis. It recently was hypothesized that PSR could induce paternal genome loss by altering histone modification in the developing *N. vitripennis* spermatids (Swim *et al.* 2012). Although PSR-induced histone modification is a plausabile hypothesis that remains to be proven, there are multiple alternative models that could explain PSR’s mode of action. For example, PSR could instead alter other chromatin-related processes, such as DNA methylation, which is known to occur at some loci in *N. vitripennis* (Park *et al.* 2011). Alternatively, transposable elements, which normally are silenced by small RNA pathways in the male and female germ lines, could be de-repressed by the presence of PSR to disrupt the chromatin state of the paternal set (Michalak 2009).

An important step in testing the aforementioned models is to assess whether PSR expresses any genes that may facilitate paternal chromatin modification. The PSR chromosome is believed to be largely heterochromatic because of its characteristic C-banding pattern (Reed 1993). Consistent with this idea, previous findings have uncovered several heterochromatin-associated sequences on the PSR chromosome, including one retrotransposable element and several satellite DNA repeats (Eickbush *et al.* 1992; McAllister and Werren 1997). In most cases, heterochromatin contains few or no genes (Grewal and Moazed 2003). Furthermore, genes that become located near centric heterochromatin through chromosomal rearrangements undergo strong transcriptional silencing (Wallrath and Elgin 1995). However, in a number of different eukaryotes some noncoding repetitive sequences located in heterochromatin, including centromere and telomere repeats, are transcribed (Kanellopoulou *et al.* 2005; Schoeftner and Blasco 2008; Volpe *et al.* 2002). These heterochromatic sequences, as well as some noncoding RNAs expressed from euchromatic loci, are known to play important roles in subnuclear organization, gene expression, and genome stability (Clemson *et al.* 2009; Meller and Rattner 2002; Plath *et al.* 2002; Sheardown *et al.* 1997). Additionally, a handful of single-copy protein coding genes have been found on the heterochromatic Y and fourth chromosomes in *Drosophila melanogaster* (Carvalho *et al.* 2001; Riddle and Elgin 2006). Thus, it is conceivable that PSR, although largely heterochromatic, expresses protein-coding genes in addition to noncoding RNAs in the wasp testis; these could facilitate modification of the paternal chromatin. Currently, no studies have directly demonstrated transcriptional activity from the PSR chromosome, and very little is known in general about genes that are carried on and expressed from supernumerary B chromosomes, or how the presence of PSR affects expression of genes from other chromosomes. It is possible that PSR’s ability to disrupt male genome transmission results from misexpression of loci located on the normal wasp chromosomes during spermatogenesis. Strong candidates for misexpression include genes encoding chromatin-associated factors, and small RNA pathway components, which play important roles in chromatin structure and genome stability (Deshpande *et al.* 2005; Megosh *et al.* 2006).

To investigate how PSR functions we have performed transcriptional profiling of *N. vitripennis* testes with and without the PSR chromosome. To our knowledge, this analysis represents the first transcriptome-based study of testis conducted in a hymenopteran insect. Our experiments demonstrate that PSR does not cause misexpression of annotated wasp chromatin-related genes, small RNA pathway genes, or TEs. However, we report the discovery of nine transcripts that are expressed uniquely from PSR. Interestingly, five of these transcripts encode putative proteins, whereas the others have poor protein-coding potential and may be long noncoding RNAs (lncRNAs). We also use these data to further annotate the wasp transcriptome and report 15.71 MB of novel transcribed regions, thus increasing the current annotation of the *N. vitripennis* genome by 53.4%. Finally, to our surprise, we found that all nine homologs of Drosophila meiosis genes that are identifiable in the wasp genome were expressed in the wasp testis, despite the lack of conventional meiosis in this sex.

## Materials and Methods

### Total RNA isolation

Total RNA was extracted using the Ambion mirVana mRNA isolation kit (Ambion/Applied Biosystems, Austin, TX). Samples were then flash frozen. The male testes were collected from 3-d-old pupae in the yellow body-red eye stage. After extraction from testes, RNA was treated with Ambion Turbo DNase (Ambion/Applied Biosystems). The quality of RNA was assessed using the Bioanalyzer 2100 (Aglient Technologies, Santa Clara, CA) and the NanoDrop 1000 UV-VIS spectrophotometer (NanoDrop Technologies/Thermo Scientific, Wilmington, DE). RNA was then prepared for sequencing using the Illumina mRNA-Seq Sample Preparation Kit (Illumina, San Diego, CA) and the Illumina HiSequation 2000 sequencer was used for sequencing paired-end–sequenced libraries (2 × 100 bp). These samples were multiplexed and run on a single lane of an Illumina flowcell. For each condition we sequenced a single sample of 80−100 pooled testes collected from multiple males.

### Poly(A)^+^ read alignment and quantification

PolyA transcriptome reads (nontrimmed) for both PSR+ (41,086,691 reads) and WT (34,468,925 reads) testes samples were processed and aligned to a reference index generated for the *Nasonia vitripennis* genome Nvit_2.0 (obtained from www.ncbi.nlm.nih.gov) and transcriptome Nvit_OGSv1.2 (obtained from www.hymenopteragenome.org/), using TopHat v2.0.8 (Trapnell *et al.* 2009). Reads were aligned using default parameters allowing up to 40 alignments per read with a maximum 2-bp mismatch. Discovery of newly transcribed regions and quantification of known isoforms and novel-transcribed features (NTRs) was performed by Cufflinks v2.0.2 (Trapnell *et al.* 2010). Differential gene expression was analyzed using the cuffdiff module of cufflinks. Sequence reads for both samples were independently aligned to annotated TEs, low complexity sequences, simple repeats, and satellites (obtained from www.hymenopteragenome.org/) using bowtie -a setting and quantified using in-house scripts.

### Discovery of PSR-specific transcripts

The poly(A)^+^ transcriptome reads for both PSR+ and WT testes samples were used to build *de novo* transcriptomes for each sample independently using Oases v0.2.08 and Velvet v.1.2.08 (Schulz *et al.*, 2012). Oases runs were performed with k-mer sizes ranging from 51 to 93 generating a total of 60,784 transcripts for the WT testes sample and 63,129 transcripts for the PSR+ testes sample. To find transcripts specific to the PSR+ sample, the transcripts produced from the aforementioned WT sample and PSR+ sample were blasted to each other, producing 2,038 PSR+ loci that had no hits against WT with an e-value cutoff of 0.1. To further filter down these transcripts, a bowtie database was produced from these transcripts and the poly(A)^+^ transcriptome reads were aligned for both samples with settings –v 0, –k 50, and –m 50 and transcript abundance was calculated as reads per million (RPM). Transcripts that had reads mapping to them from the WT sample were excluded, and we required that the PSR-specific transcripts were abundantly expressed and had at least 50 reads mapping to them. This stringent filtering resulted in nine PSR-specific transcripts.

### Discovery of NTRs

To search for NTRs, we used the current assembly of the *N. vitripennis* genome (Nvit_2.0_scaffolds downloaded from http://www.hymenopteragenome.org) that contains 6169 contigs and is 295 MB in size, almost twofold larger than the genome of *Drosophila melanogaster*. This existing genomic annotation, which contains 18,833 genes and 18,923 transcripts, was used as a starting point for our analysis (Munoz-Torres *et al.* 2011; Werren *et al.* 2010a). Sequence reads from both testes samples, HiSeq2000 paired-end–sequenced libraries (2 × 100 bp), were used to build *de novo* transcriptomes (genome supplied and no transcriptome supplied) for each sample, using cufflinks v2.0.2 (Trapnell *et al.* 2012). Transcript annotation files in GTF format produced by cufflinks for each individual library were combined and cross-referenced with known genes using the cuffmerge module of cufflinks. This resulted in the identification of 2293 new transcribed regions. The coding potential of NTRs was assessed using the frame finder tool in ESTate (Expressed Sequence Tag Analysis Tools Etc) package (http://www.ebi.ac.uk/~guy/estate/). Protein domains were predicted using the stand-alone InterProScan package (iprscan) (Zdobnov and Apweiler 2001).

### Fluorescence *in situ* hybridization and chromosome imaging

The following primers were designed commercially (IDT, Inc.) and conjugated at the 5′ terminus with either Cy5 or Cy3: PSR Locus 317: 5′-TGT AAC TGG AAA AGG AAA ATG TAT TAT TGA-3′; PSR Locus 1539: 5′-AGA ATT ATA ATA TAG TTA GCT GGA CAA TTC-3′; PSR Locus 5885: 5′-TTC GTG TGT GTA TAA AAT TAT ATA TTC TCA AA-3′; Wasp Locus TCONS_00014084: 5′-AAT TTT GTG AAT TTT GGT GTC TCC ATC-3′; and Wasp Locus TCONS_00004522: 5′-TCT AAT CAA ACG TGA ATT TGG TGT TTT TAA-3′. These probes were hybridized to fixed testes taken from male pupae in the yellow body-red eye stage, according to a previously described protocol (Swim *et al.* 2012). Slides were prepared by mounting samples in Vectashield with DAPI (Vector Labs, Inc.). Chromosome images were collected on an Olympus IX81 epifluorescence microscope and ImagePro 6.3 imaging software. The images were processed with Adobe Photoshop CS5 version 12.

## Results

### Identification of newly transcribed regions through analysis of the *N. vitripennis* testis transcriptome

To explore the RNA expression profile of the *N. vitripennis* testis, we isolated and analyzed poly(A)^+^ RNAs from single samples of pooled, whole testes of both WT *AsympC* and PSR-carrying *AsymC* male wasps. From a total of 80,020,380 reads produced from both conditions, 74,859,927 (93.55%) mapped to the wasp genome (Supporting Information, Table S1). Using these data, we first explored the wasp genome for nonannotated transcribed features to generate a more complete transcriptome. The transcriptomes from WT and PSR-containing testes were used to produce a combined gene set, which included 18,924 known transcripts and 14,857 new isoforms of previously annotated *N. vitripennis* genes. These new isoforms originate from 40.3% of the previously annotated genes, and added a total of 150,189 novel exons (File S1). We also discovered 2293 novel transcribed regions (NTRs) that do not match known annotations. These NTRs were produced from 1979 loci with a total of 4450 novel exons that do not overlap annotated *N. vitripennis* transcription units (File S2, Table S2). The new transcriptome assembly confirmed 52,516 of 84,899 exon junctions derived from *N. vitripennis* transcripts (61.9%) and defined 64,743 novel exon junctions, significantly increasing the splicing complexity of the *N. vitripennis* transcriptome. A total of 63,013 (97.3%) newly discovered junctions belong to new isoforms of *N. vitripennis* genes, and 1730 (2.7%) originate from NTR transcripts. To put these findings into perspective, the previous *N. vitripennis* transcriptome covers 29.43 MB of genomic sequence (9.98% of the assembled genome). The new isoforms of previously annotated genes from this analysis increase the genomic sequence coverage to 42.94 MB, and the NTRs add another 2.20 MB. Thus, the transcripts identified from our poly(A)^+^ RNA samples result in an increase of transcribed sequence from 29.43 MB to 45.14 MB (a 53.4% increase), resulting in a total of 15.3% of the genome being transcribed into poly(A)^+^ RNAs in the *N. vitripennis* testis. This level is similar to that found in the *D. melanogaster* testis (Adams *et al.* 2000).

To attain a better understanding of what the identified NTRs may encode, we conducted homology searches against the NCBI nonredundant protein database and identified significant hits for 770 (33.58%) NTRs (Table S3). For 404 NTRs (52.46% of hit-producing NTRs), the best blast hits were against *N. vitripennis* proteins, suggesting that they may represent paralogs of previously annotated *N. vitripennis* genes. In total, 66.41% (1523) of NTRs discovered had no significant blast hits, suggesting these may be novel genes specific to *N. vitripennis* (Table S4, File S3). Only four of the 1523 NTRs with no blast hits have potential open reading frames (ORFs) longer than 200 amino acids (Table S5). All four of these potential ORFs contain a potential coiled-coiled domain but no other identifiable functional domains. It is possible that most of the NTRs with no blast hits are noncoding RNAs, although we cannot rule out the possibility that some encode small proteins. To create a subset of relatively highly expressed noncoding NTRs, we identified the NTRs that are expressed with at least 10 fragments per kilobase of exon per million fragments mapped in at least one of the two samples, are at minimum 200 base pairs long, and have no significant blast hits or ORFs longer than 200 amino acids. These criteria produced a total of 202 highly expressed, potentially noncoding transcripts (Table S6, File S4).

### Identification of PSR-expressed transcripts

A primary goal of this study was to identify any transcripts that are specifically expressed from the PSR chromosome in the testis. To identify PSR-specific transcripts, we stringently excluded all transcripts that had any matches to WT transcripts. In total, we discovered nine expressed transcripts (10−1163 RPM) that were exclusively identified in the PSR sample (see *Materials and Methods* for details). All of the remaining PSR-specific transcripts, which had RPMs of <10 and <50 map-able reads, were excluded based on their low expression. Additionally, there were 343 transcripts that had only one WT read that mapped to them, but these transcripts also had very few PSR reads that mapped to them as well. For example, the greatest number of PSR reads for those 343 transcripts was 14, compared with 7952 reads for Locus_4317 (the greatest PSR-specific transcript). Therefore, the transcripts that had any WT reads mapping to them and also very few PSR reads were excluded from our analyses. We chose to focus our study on highly expressed PSR-specific transcripts because they are much stronger candidates for paternal genome alteration than low expressed transcripts. Additionally, as expected we found no transcripts expressed above background levels (*i.e.*, more than 10 map-able reads or greater than 2RPMs) that are specific to the WT sample (*i.e.*, not expressed in the PSR+ sample).

The lengths of these PSR-specific transcripts ranged from 575 bp to 1483 bp, with an average length of 948 bp. None of these transcripts were conserved at the nucleotide level to any sequences in the nucleotide collection “nr/nt” NCBI blastn database. However, all of these transcripts contained putative ORFs, but many of them were small; the longest predicted ORF length was 245aa (Table S7). To explore what these transcripts could potentially encode, we blasted translated nucleotide sequences against the nonredundant protein database (blastx). Four of the nine transcripts had significant blastx hits (E-value < 0.01; Table S7). Of these transcripts, three (Loci 5885, 2141, and 9281) had top hits to annotated hypothetical *N. vitripennis* proteins. Two had limited homology to either *Nasonia* hypothetical protein LOC100680517 (query coverage 24%, E-value 6e-07, chromosomal location unknown) or to *Nasonia* hypothetical protein LOC100678674, an F-Box domain-containing gene (query coverage 55%, E-value 2e-15, located on chromosome 4). These PSR-specific transcripts are likely paralogs of these *N. vitripennis* genes. The third transcript (Locus 2141) had extensive homology to two genes, including a tnp2 family transposase-like gene, *Nasonia* hypothetical protein LOC100678713 (query coverage 94%, E-value 2e-76, chromosomal location unknown), and predicted hypothetical protein LOC100678738 (query coverage 99%, E-value 2e-70, located on chromosome 2), and is, therefore, a likely paralog of these genes.

The fact that these *N. vitripennis* paralogs for two of the PSR-specific genes are located on different chromosomes argues that the PSR regions producing these transcripts likely do not originate from a single chromosomal region. Additionally, one transcript (Locus 4317) has some homology to a hypothetical protein from *Caenorhabditis briggsae* (Query coverage 27%, E-value 7e-05). Of the transcripts that did not have any significant blastx hits (5/10), three (Loci 1539, 4656, and 8628) were found to have poor coding potential (see *Materials and Methods*), primarily because of very short predicted ORFs and a lack of extensive homology to known genes (Kong *et al.* 2007). On the basis of these characteristics, these sequences may belong to the long (>200 bp) class of noncoding RNAs. The two final sequences (Loci 5643 and 5794) have no homology to known genes, but each contains a putative conserved protein domain (RRM-SF domain accession number cd00590 for Locus 5643 and Coatomer-E domain accession number pfam04733 for Locus 5794) within their predicted ORFs.

**Figure 1:**
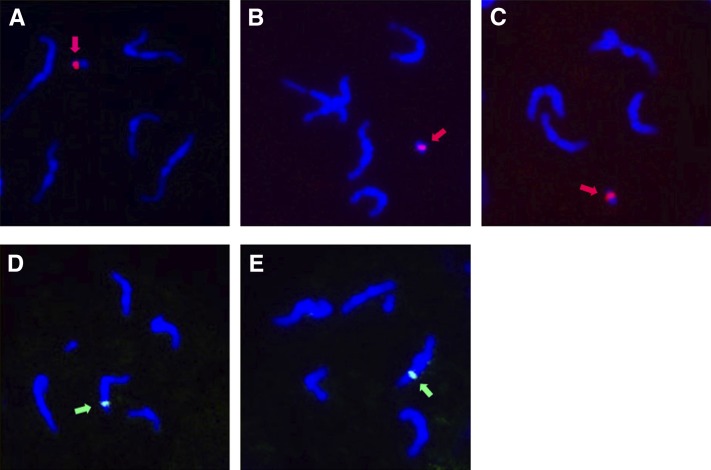
Chromosomal locations of PSR-specific and normal chromosome transcripts obtained from RNA-seq by using DNA *fluorescence in situ hybridization*. (A−C) Three probes, each corresponding to one of three transcripts (names, respectively) present only in the PSR+ testis, localize specifically to the PSR chromosome (red regions, indicated by red arrows). (D, E) Probes corresponding to two different transcripts present in both control and PSR+ testes highlight single regions on a single normal chromosome.

To determine which chromosome the PSR-specific transcripts arise from, we mapped the corresponding chromosomal locations of the three most abundant transcripts (Loci 4317, 5885, and 1539) by using DNA *fluorescence in situ hybridization* (see *Materials and Methods*; Table S7). These transcripts included the two sequences with homology to either the *C. briggsae* hypothetical protein or the *N. vitripennis* hypothetical protein (LOC100680517) and one putative lncRNA (LOC1539). Each of these sequences localized to a single region on the PSR chromosome, and they did not hybridize to any of the normal wasp chromosomes (Figure 1, A−C). We also mapped two potentially noncoding transcripts that were highly expressed in both WT and PSR-containing testis and, therefore, are likely to be derived from the normal chromosomes. Each of these sequences mapped to single regions on different wasp chromosomes but not to PSR (Figure 1, D and E). These patterns confirm the results of our transcriptional (RNA-Seq) analyses, demonstrating the specificity of these sequences to either PSR or the normal chromosomes.

### Expression of chromatin-associated genes in the presence of PSR

**Figure 2:**
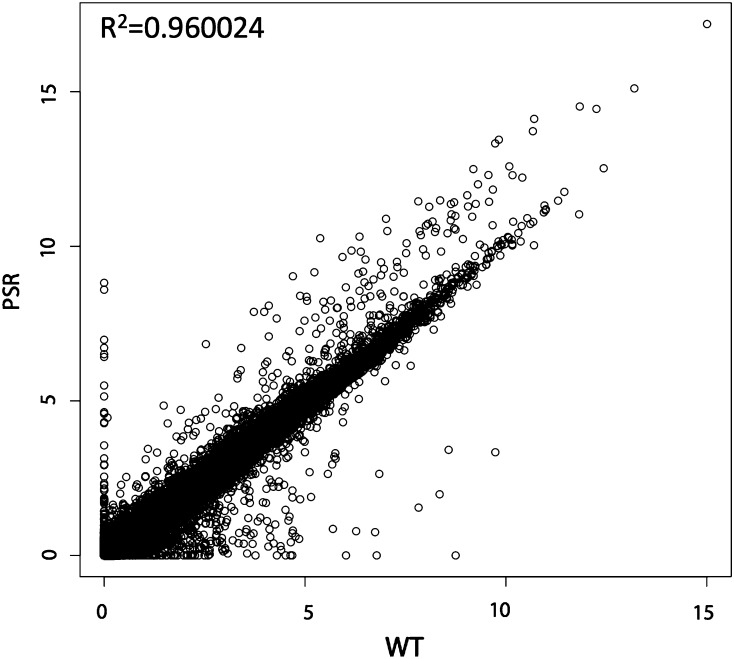
Scatter plot of PSR *vs.* WT testes with coefficient of determination. Scatter plot of fragments per kilobase of exon per million fragments mapped values for genes and NTRs comparing expression values for PSR (Y-axis) and WT (X-axis) testes. The coefficient of determination (R^2^) is displayed in top left.

Another goal of this study was to identify any genes in the normal wasp genome that are misexpressed and to assess whether TEs become transcriptionally hyperactive, in the presence of PSR. First, using our comprehensive testis transcriptome, we calculated the expression levels for all previously annotated genes and our NTRs, and observed a strong correlation between the WT and PSR-containing testis samples (Pearson Correlation Coefficient 0.962; Figure 2, Table S8, and Table S9). Thus, expression of the vast majority of wasp genes in the testis is not affected by PSR. To further investigate the genes that were misexpressed in the presence of PSR, we performed an over/underrepresentation analysis, which revealed several patterns. First, we found a total of 199 previously annotated genes and NTRs that are overrepresented in the PSR testis and 345 previously annotated genes and NTRs that are underrepresented in the PSR testes compared with WT testes (*P* < 0.05; Table S10 and Table S11). A gene ontology analysis revealed that the majority of overexpressed genes are involved in cellular carbohydrate biosynthesis, protein polymerization, and cellular protein complex assembly. The majority of genes that were under-represented in the PSR testes are involved in chitin metabolism, aminoglycan metabolism, and polysaccharide metabolism. There were also smaller numbers of genes in other gene ontology categories that were also up- or down-regulated in PSR testes and these may also be important for the role PSR plays (Table S12 and Table S13).

Second, we found no evidence for misexpression of homologs of *D. melanogaster* genes that are involved in different aspects of chromatin structure and stability in the presence of PSR. These genes include putative histone acetyltransferases (n = 13), histone methyltransferases (n = 28), histone deacetylases (n = 6), histone demethylases (n = 16), DNA methylase 1 (Dnmt1), and the heterochromatin protein 1 (Table S14). Additionally, we found normal expression levels for genes belonging to the small interfering (si-) RNA, micro- (mi-) RNA, and Piwi-interacting (pi-) RNA pathways (Table S14). Finally, we found no evidence of TE overexpression in the PSR-containing testis. Surprisingly, the number of reads that mapped to annotated TEs, low complexity sequences, simple repeats, and satellites was extremely low for both samples at (0.0022% for PSR and 0.0029% for WT, of the total reads produced), suggesting that transcription of these genes is negligible in the testes (Table S15). We did detect very few reads originating from the PSR-specific NAsonia Transposable Element (*NATE*) only in the PSR sample and not the WT sample, indicating that this TE is transcribed from the PSR chromosome (McAllister 1995; McAllister *et al.* 2004). Finally, protamine-encoding genes remain to be identified in *N. vitripennis*; therefore, we were unable to determine if the expression levels of these genes are altered by PSR.

### Expression of meiosis-related genes in the *N. vitripennis* testis

In *N. vitripennis*, male meiosis occurs as a modified form of mitosis (Whiting 1968), which is why we expected to detect few if any meiosis-related genes in the testis. To our surprise, we found that of the nine putative orthologs of known *D. melanogaster* meiotic genes that were identifiable in the wasp genome, all nine were expressed at high levels in the wasp testis (Table S16). At least one of these genes, *meics* (*meiotic central spindle*) may have a meiosis-specific role in *D. melanogaster* (Panzera *et al.* 2001), whereas many of the other genes are known to have additional roles in mitosis or other processes unrelated to meiosis within spermatogenesis.

## Discussion

In this study we performed transcriptional profiling of the *N. vitripennis* testis to begin to understand how the selfish B chromosome, PSR, modifies the paternal nucleus for destruction. A primary goal was to identify any coding or noncoding genes that are expressed from PSR and that may play a role in this phenomenon (discussed herein). However, an additional outcome was the discovery of 15.71 Mbp of NTRs, which raises the known amount of transcribed regions in the *N. vitripennis* genome by 53.0%. This dataset provides the first complete account of global gene expression in the *N. vitripennis* testis and can be used as a resource for future studies aimed at investigating developmental processes tied to the male germ line in hymenopterans.

One interesting pattern borne out of this work is our finding that all nine orthologs of Drosophila meiosis-related genes present in the *N. vitripennis* genome are expressed in the male germline of this species. This finding is contrary to our prediction that meiosis genes would not be expressed in the wasp testes because of the fact that meiosis in the male sex is a modified mitosis (Whiting 1968). In flies, eight of these genes are known to function in additional non-meiotic processes. For example, *mei-w68* is involved in initiation of meiotic recombination but also operates in S phase and mitosis similarly to Topoisomerase 2 (Top2), which catenates and decatenates DNA (McKim and Hayashi-Hagihara 1998). Another gene, *meiosis I arrest* (*mia*), facilitates the G2/M transition of meiosis I and also the onset of spermatid differentiation (Lin *et al.* 1996; White-Cooper *et al.* 1998). Thus, it is possible that these genes only perform their non-meiotic functions in the wasp male germ line. In contrast, *meiotic central spindle* (*meics*) may be restricted to meiosis-related functions in flies (Panzera *et al.* 2001). What functions might the ortholog of this or other meiosis-specific genes serve in the wasp testis? Haplodiploidy is believed to have evolved from a diploid state with traditional meiosis in the male sex (Bull 1981). It is intriguing to speculate that *meics* and perhaps others may have evolved to perform new functions in the wasp testis. These new functions, in turn, may have helped to facilitate the transition from a normal meiosis in diploids involving two homologous chromosome sets to a mitosis-like meiosis in hymenopterans.

### PSR expresses transcripts with coding and noncoding potential in the wasp testis

Although the vast majority of B chromosomes are largely heterochromatic (Jones 1995; Jones *et al.* 2008), little is known about the specific sequence elements that they contain, and even less is known about their potential for gene expression. Our analyses have led to the identification of nine transcripts that are uniquely expressed from the PSR chromosome in the wasp testis. Four have strong potential to code for proteins. Three of these putative proteins (Loci 5885, 2141, and 9281) are at least partly homologous to hypothetical or uncharacterized *N. vitripennis* proteins, while the other (Locus 4317) partially matches a hypothetical protein found in *C. briggsae*. Notably, one transcript (Locus 2141) that is homologous to a *N. vitripennis* sequence appears to encode a transposase-like protein. We did detect low PSR-exclusive expression (*i.e.*, no expression in the WT condition) from the previously identified retrotransposable element, NATE, which is located on the PSR chromosome (McAllister 1995; McAllister and Werren 1997), suggesting that PSR is actively transcribing TEs and that these may play a role in its mechanism. However, the lack of additional TE-associated transcripts uniquely present in the PSR-containing testis indicates that the population of expressed TEs carried on the PSR chromosome is very similar to that present within the normal wasp genome. In general, the presence of PSR has very little effect on TE expression in the testis.

Of the five remaining PSR-specific transcripts that we detected, three (Loci 1539, 4656, and 8628) have poor coding potential while two (Loci 5643 and 5794) have low but significant coding potential. Thus, there is a strong possibility that at least some of these sequences are lncRNAs. Interestingly, we mapped the three most highly abundant PSR-specific transcripts, which includes one putative lncRNA (Locus 1539) and two transcripts with strong coding potential (Loci 4317 and 5885), to the subchromosomal level by using DNA fluorescence *in situ* hybridization, and in doing so, we found that each of these three transcripts correspond to a single, unique region on the PSR chromosome. This finding has several implications. First, it is possible that either these transcripts are expressed directly from the mapped loci or they are instead expressed from small euchromatic regions that are distinct from the mapped loci and located within the larger heterochromatic context of the PSR chromosome. Either scenario is possible, given that (1) transcripts are known to be expressed directly from heterochromatic sequences such as telomere repeats (Schoeftner and Blasco 2008) and (2) a handful of genes are located within and expressed from heterochromatic chromosomal regions, including the pericentromeric region of the X (Tautz *et al.* 1988) as well as the largely heterochromatic Y and fourth chromosomes in *D. melanogaster* (Koerich *et al.* 2008; Riddle and Elgin 2006; Vibranovski *et al.* 2008). A second interesting implication pertains specifically to the transcripts with high protein-coding potential that were mapped to PSR. Future studies will be required to directly test which, if any, of these transcripts are translated in the testis. However, if they are, indeed, translated and also are transcribed from high-copy heterochromatic sequences, then they would represent a rare class of complex repeats that encode proteins. One other complex repeat, Stellate, is located in multiple, tandemly repeated copies on the distal end of the X pericentromeric heterochromatin of the *D. melanogaster* X chromosome (Palumbo *et al.* 1994; Shevelyov 1992; Tritto *et al.* 2003). Normally in the testis, this locus is transcriptionally silenced through the piRNA pathway by a Y-linked locus, Suppressor of Stellate, or Su(Ste) (Nishida *et al.* 2007). In the absence of Su(Ste), the Stellate repeats produce a transcript that encodes a Casein Kinase-like protein. Expression of this protein leads to formation of crystalline aggregates in the testis and male sterility (Bozzetti *et al.* 1995).

Could any of the PSR-expressed transcripts facilitate modification of the paternal half of the wasp genome, and if so, how? None of the predicted ORFs of these transcripts encodes proteins with specific motifs or overall topologies that strongly suggest ties to chromatin dynamics. However, it is reasonable to imagine that one or more of these proteins, if produced, may differentially associate with the paternal chromatin in some deleterious manner. Large differences in chromatin composition, such as high amounts of euchromatin in the normal wasp genome, may attract such proteins more than the heterochromatic PSR chromosome, resulting ultimately in hypercondensation of the paternal set and concomitant exemption of PSR from this fate.

A second, intriguing possibility is that PSR-expressed lncRNAs may facilitate modification of the paternal set. This idea stems from the fact that a number of lncRNAs, including *roX1* and *roX2* in *D. melanogaster* and *Xist* in mouse and human, are known to facilitate chromatin-based processes. In flies, both *roX1* and *roX2* are expressed from a locus on the X chromosome (Amrein and Axel 1997). These lncRNAs are integral components of the dosage compensation complex, which forms only in the male sex and localizes broadly across the euchromatic part of the single X chromosome (Franke and Baker 1999). The *roX* RNAs may serve to guide the dosage compensation complex to the X, where it can facilitate remodeling of the X euchromatin into a state that is more accessible for transcriptional machinery (Gu *et al.* 1998; Hilfiker *et al.* 1997; Lucchesi *et al.* 2005). In mammals, *Xist*, which also is X-linked, becomes expressed from one of the two X chromosomes at random during early development (Brown *et al.* 1991; Kay *et al.* 1993; Marahrens *et al.* 1998; Penny *et al.* 1996). In conjunction with other lncRNAs, *Xist* acts *in cis* to transform the X chromosome into a transcriptionally silent, heterochromatin state (Chaumeil *et al.* 2006; Heard 2005; Navarro *et al.* 2005; Sun *et al.* 2006). Therefore, in one scenario, PSR-expressed transcripts could operate *in trans*, associating with paternal euchromatin and either blocking normal chromatin enzyme activities or inducing abnormal enrichment of chromatin factors. Alternatively, PSR-expressed lncRNAs may associate specifically with the PSR chromosome as a way of protecting it from a second activity that acts to modify the paternal chromatin. Determining the localization patterns of these transcripts at the subnuclear level in the testis and early embryo will be important for testing these and other ideas.

**Figure 3:**
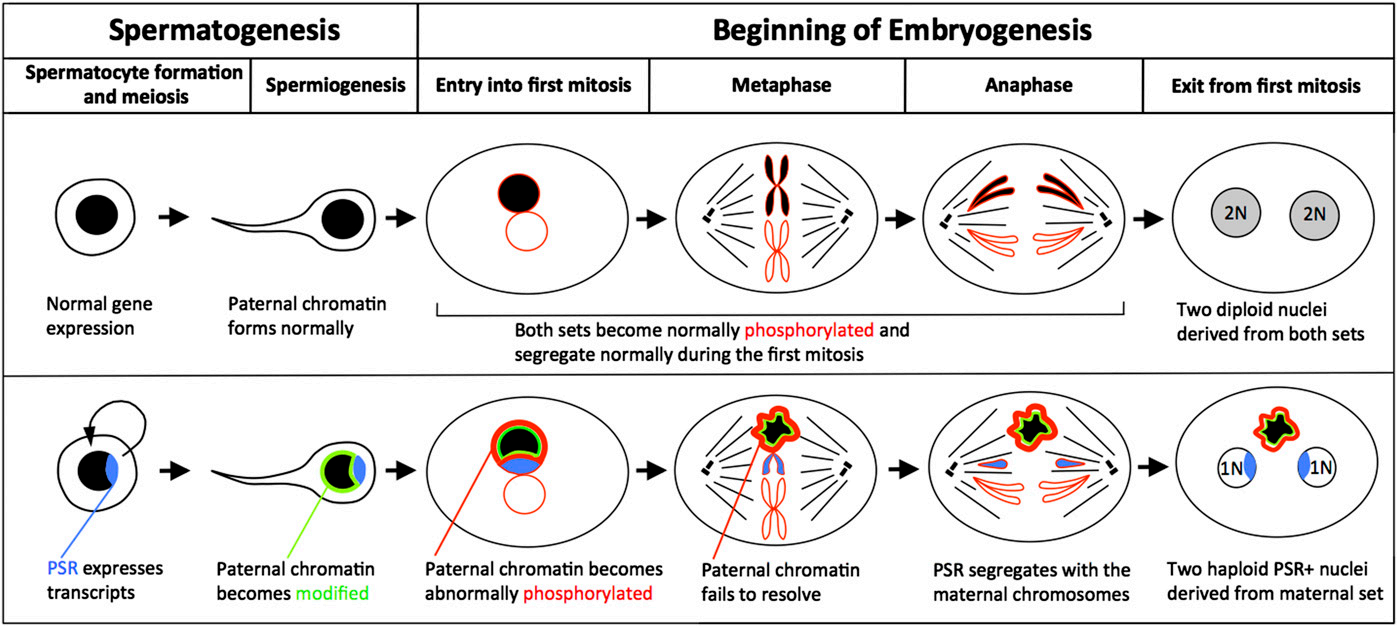
Model for initial alteration of the paternal genome by PSR as suggested by testis transcriptome profiling. PSR-specific transcripts are expressed during spermatogenesis. These transcripts or their encoded proteins facilitate an unknown modification of the paternal chromatin that disrupts its normal behavior during the first mitotic division of the embryo. PSR, however, spares itself this fate and is transmitted with the viable maternal set. The paternal set is lost, converting what should become a diploid female embryo into a haploid male, the PSR-transmitting sex.

Our studies have revealed that PSR expresses both potentially coding and noncoding transcripts, despite its largely heterochromatic composition. We propose that these transcripts could play a role in the ability of PSR to destroy the paternal half of the genome and/or protect the PSR chromosome, in order to facilitate its transmission to males (Figure 3). That said, other transcription-dependent and independent models exist to explain selfish PSR behavior (Werren and Stouthamer 2003); our study is a first step toward investigating the possibilities at a genomic level. The analysis of poly(A)^+^ transcripts presented here will complement future experiments that explore nonadenylated transcripts and small RNAs in the wasp testis to provide a more complete transcriptional profile of gene expression from PSR and WT chromosomes.

## Supplementary Material

Supporting Information
