# Supplementary material for: Transcriptome Profiling of Nasonia vitripennis Testis Reveals Novel Transcripts Expressed from the Selfish B Chromosome, Paternal Sex Ratio
Source: G3 (Bethesda). 2013 Sep 1;3(9):1597–605. doi: 10.1534/g3.113.007583 (PMC3755920; doi:10.1534/g3.113.007583)
Supplement: Supporting Information [file supp_3_9_1597__index.html]

Transcriptome Profiling of Nasonia vitripennis Testis Reveals Novel Transcripts Expressed from the Selfish B Chromosome, Paternal Sex Ratio — Supporting Information 

# Transcriptome Profiling of *Nasonia vitripennis* Testis Reveals Novel Transcripts Expressed from the Selfish B Chromosome, Paternal Sex Ratio

## Supporting Information for Akbari *et al.*, 2013

**Files in this Data Supplement:**

- Supporting Information - Files S1-S5 and Tables S1-S16 (PDF, 385 KB)
- File S5 - Methods (PDF, 350 KB)
- File S1 - Novel isoforms of Annotated Genes GTF file (.zip, 2 MB)
- File S2 - Novel Transcribed Regions GTF file (.zip, 78 KB)
- File S3 - Novel Transcribed Regions with no Blastx hits GTF file (.zip, 47 KB)
- File S4 - Expressed Non-coding NTRs GTF file (.zip, 7 KB)
- Table S1 - Mapping Statistics (.xlsx, 11 KB)
- Table S2 - Novel Transcribed regions (NTR) fasta file (.xlsx, 983 KB)
- Table S3 - Blastx results for NTRs (.xlsx, 313 KB)
- Table S4 - NTRs with no significant blast hits fasta file (.xlsx, 616 KB)
- Table S5 - NTRs with Coding potential fasta file (.xlsx, 10 KB)
- Table S6 - Non-coding Novel Transcribed regions (NTR) fasta file (.xlsx, 63 KB)
- Table S7 - Transcripts specific to PSR (.xlsx, 15 KB)
- Table S8 - Gene Expression (.xlsx, 2 MB)
- Table S9 - Transcript Expression (.xlsx, 2 MB)
- Table S10 - PSR Overrepresented genes and NTRs (.xlsx, 35 KB)
- Table S11 - PSR Underrepresented genes and NTRs (.xlsx, 55 KB)
- Table S12 - PSR Ontology Overrepresentation analysis (.xlsx, 19 KB)
- Table S13 - WT Ontology Overrepresentation analysis (.xlsx, 17 KB)
- Table S14 - Expression Patterns for chromatin remodeling enzymes and Small RNA processing genes (.xlsx, 852 KB)
- Table S15 - Transposable elements, simple repeats, satellites, and low complexity sequence expression profiles (.xlsx, 111 KB)
- Table S16 - Conserved meiosis related genes expression (.xlsx, 25 KB)
