# Supplementary material for: Transcriptome Profiling of Nasonia vitripennis Testis Reveals Novel Transcripts Expressed from the Selfish B Chromosome, Paternal Sex Ratio
Source: G3 (Bethesda). 2013 Sep 1;3(9):1597–605. doi: 10.1534/g3.113.007583 (PMC3755920; doi:10.1534/g3.113.007583)
Supplement: Supporting Information [file supp_g3.113.007583_007583SI.pdf]

G3 · Genes | Genomes | Genetics

Transcriptome Profiling of *Nasonia vitripennis* testis reveals Novel Transcripts Expressed from the selfish B chromosome, Paternal Sex Ratio

Omar S. Akbari[1,*], Igor Antoshechkin[1], Bruce A. Hay[1], Patrick M. Ferree[2,*]

1. Division of Biology, MC156-29, California Institute of Technology, Pasadena, CA 91125, USA

2. W. M. Keck Science Department of Claremont McKenna, Pitzer, and Scripps Colleges 925 N. Mills Avenue, Claremont, CA 91711, USA

*Correspondence:

Patrick Ferree: pferree@kecksci.claremont.edu
Omar S. Akbari: oakbari@caltech.edu

DOI: 10.1534/g3.113.007583

File S1
Novel isoforms of Annotated Genes GTF file

File S2
Novel Transcribed Regions GTF file

File S3
Novel Transcribed Regions with no Blastx hits GTF file

File S4
Expressed Non-coding NTRs GTF file

Files S1-S4 are available for download at http://www.g3journal.org/lookup/suppl/doi:10.1534/g3.113.007583/-/DC1.

File S5

Methods

Total RNA Isolation

Total RNA was extracted using the Ambion mirVana mRNA isolation kit (Ambion/Applied Biosystems, Austin, TX). Samples were then flash frozen. The male testes were collected from 3-day-old pupae in the yellow body-red eye stage. Following extraction from testes, RNA was treated with Ambion Turbo DNase (Ambion/Applied Biosystems, Austin, TX). The quality of RNA was assessed using the Bioanalyzer 2100 (Aglient Technologies, Santa Clara, CA) and the NanoDrop 1000 UV-VIS spectrophotometer (NanoDrop Technologies/Thermo Scientific, Wilmington, DE). RNA was then prepared for sequencing using the Illumina mRNA-Seq Sample Preparation Kit (Illumina San Diego, CA) and the Illumina HiSeq 2000 sequencer was used for sequencing paired-end–sequenced libraries (2 x 100bp). These samples were multiplexed and run on a single lane of an Illumina flowcell. For each condition we sequenced a single sample of 80-100 pooled testes collected from multiple males.

Poly(A)+ Read Alignment and Quantification

PolyA transcriptome reads (non-trimmed) for both PSR+ (41,086,691 reads) and WT (34,468,925 reads) testes samples were processed and aligned to a reference index generated for the *Nasonia vitripennis* genome Nvit_2.0 (obtained from www.ncbi.nlm.nih.gov) and transcriptome Nvit_OGSv1.2 (obtained from www.hymenopteragenome.org/), using TopHat v2.0.8 (Trapnell et al., 2009). Reads were aligned using default parameters allowing up to 40 alignments per read with a maximum 2bp-mismatch. Discovery of newly transcribed regions and quantification of known isoforms and NTRs was performed by Cufflinks v2.0.2 (Trapnell et al., 2010). Differential gene expression was analyzed using the cuffdiff module of cufflinks. Sequence reads for both samples were independently aligned to annotated TEs, low complexity sequences, simple repeats and satellites (obtained from www.hymenopteragenome.org/) using bowtie -a setting and quantified using in-house scripts.

Discovery of PSR-specific Transcripts

The poly(A)+ transcriptome reads for both PSR+ and WT testes samples were used to build *de novo* transcriptomes for each sample independently using Oases v0.2.08 and Velvet v.1.2.08 (Schulz et al., 2012; Zerbino, 2010). Oases runs were performed with k-mer sizes ranging from 51 to 93 generating a total of 60,784 transcripts for the wild type testes sample and 63,129 transcripts for the PSR+ testes sample. To find transcripts specific to the PSR+ sample, the transcripts produced from the WT sample and PSR+ sample above were blasted to each other, producing 2,038 PSR+ loci that had no hits against WT with an e-value cutoff of 0.1 To further filter down these transcripts, a bowtie database was produced from these transcripts and the poly(A)+ transcriptome reads were aligned for both samples with settings –v 0, -k 50 and –m 50 and transcript abundance was calculated as Reads Per Million (RPM). Transcripts that had reads mapping to them from the WT sample were excluded and we required that the PSR specific transcripts were abundantly expressed and had at least 50 reads mapping to them. This stringent filtering resulted in 9 PSR specific transcripts.

Discovery of NTRs

To search for novel transcribed features (NTRs), we used the current assembly of the *N. vitripennis* genome (Nvit_2.0_scaffolds downloaded from http://www.hymenopteragenome.org) that contains 6,169 contigs and is 295 MB in size, ~2-fold larger than

the genome of *Drosophila melanogaster*. This existing genomic annotation, which contains 18,833 genes and 18,923 transcripts, was used as a starting point for our analysis (Munoz-Torres et al., 2011; Werren et al., 2010a). Sequence reads from both testes samples, HiSeq2000 paired-end–sequenced libraries (2 x 100bp), were used to build *de novo* transcriptomes (genome supplied and no transcriptome supplied) for each sample, using cufflinks v2.0.2 (Trapnell et al., 2012). Transcript annotation files in GTF format produced by cufflinks for each individual library were combined and cross-referenced with known genes using the cuffmerge module of cufflinks. This resulted in the identification of 2,293 new transcribed regions. The coding potential of NTRs was assessed using the frame finder tool in ESTate (Expressed Sequence Tag Analysis Tools Etc) package (http://www.ebi.ac.uk/~guy/estate/). Protein domains were predicted using the stand alone InterProScan package (iprscan) (Zdobnov E.M. and Apweiler R. *"InterProScan - an integration platform for the signature-recognition methods in InterPro"* Bioinformatics, 2001, **17(9)**: p. 847-8.).

Fluorescence in situ hybridization **(FISH) and chromosome imaging**

The following primers were designed commercially (IDT, Inc.) and conjugated at the 5' terminus with either Cy5 or Cy3: PSR Locus 317 – 5'-TGT AAC TGG AAA AGG AAA ATG TAT TAT TGA-3'; PSR Locus 1539 – 5'-AGA ATT ATA ATA TAG TTA GCT GGA CAA TTC-3'; PSR Locus 5885 – 5'-TTC GTG TGT GTA TAA AAT TAT ATA TTC TCA AA-3'; Wasp Locus TCONS_00014084 – 5'-AAT TTT GTG AAT TTT GGT GTC TCC ATC-3'; Wasp Locus TCONS_00004522 – 5'-TCT AAT CAA ACG TGA ATT TGG TGT TTT TAA-3'. These probes were hybridized to fixed testes taken from male pupae in the yellow body-red eye stage, according to a previously described protocol (Swim et al. 2012). Slides were prepared by mounting samples in Vectashield with DAPI (Vector Labs, Inc.). Chromosome images were collected on an Olympus IX81 epifluorescence microscope and ImagePro 6.3 imaging software. The images were processed with Adobe Photoshop CS5 version 12.

Tables S1-S16 are available for download at http://www.g3journal.org/lookup/suppl/doi:10.1534/g3.113.007583/-/DC1.

Table S1 Mapping Statistics. Mapping statistics for both PSR+ and WT testes samples indicating the total reads which map to junctions, exons, to multiple locations, uniquely and total locations mapped.

Table S2 Novel Transcribed regions (NTR) fasta file.

Table S3 Blastx results for NTRs.

Table S4 NTRs with no significant blast hits fasta file.

Table S5 NTRs with Coding potential fasta file.

Table S6 Non-coding Novel Transcribed regions (NTR) fasta file.

Table S7 Transcripts specific to PSR. A list of transcripts specific to PSR including relative expression levels in reads per million (RPM).

Table S8 Gene Expression. Gene expression profile for Annotated Genes and NTR's. 20,813 total - 18,833 Annotated Genes and 1,980-NTRs.

Table S9 Transcript Expression. Gene expression profile for Annotated transcripts and NTR transcripts. 21,216 total -18,923-Annotated transcripts and 2293 NTRs.

Table S10 PSR Overrepresented genes and NTRs. A list of 199 genes and NTRs significantly overrepresented in the PSR testes compared to the wildtype testes. Terminology: gene_id: gene ID; locus: Chromosomal start-stop positions; WT FPKM: FPKM from the WT sample; PSR FPKM: FPKM from the PSR sample; log2(fold change): Fold change of the log(2) FPKM data. Test stat: The value of the test statistic used to compute significance of the observed change in FPKM, p value: The uncorrected *p*-value of the test statistic, q value: The FDR-adjusted *p*-value of the test statistic.

Table S11 PSR Underrepresented genes and NTRs. A list of 345 genes and NTRs significantly overrepresented in the WT testes compared to the PSR testes. For terminology see supplementary table 10 legend.

Table S12 PSR Ontology Overrepresentation analysis. A list of the genes overrepresented in the PSR testes compared to the wildtype testes. Terminology: GOBPID: Gene Ontology Biological Process Identification number; GOMFID: Gene Ontology Molecular Function Identification number; Pvalue: p value given by the hypergeometric test ($p<0.01$); OddsRatio: of odds that a GO term is enriched in the selected category; ExpCount: expected number of transcripts found associated with the GO term for enrichment; Count: real number of transcripts found associated with the GO term; Size: population size of transcripts found associated with the GO term within the analysis; Term: Gene Ontology Biological Process description term; Inf: Infinite value.

Table S13 WT Ontology Overrepresentation analysis. A list of the genes overrepresented in the WT testes compared to the PSR testes. For terminology see legend for Table S12 legend above.

Table S14 Expression Patterns for chromatin remodeling enzymes and Small RNA processing genes. A list of expression patterns for Putative histone Deacetylases, Putative histone Demethylases, Putative Acetyltransferases, DNA methyltransferases and small RNA processing and piRNA related genes for both PSR and WT conditions.

Table S15 Transposable elements, simple repeats, satellites, and low complexity sequence expression profiles.

Table S16 Conserved meiosis related genes expression. A list of expression patterns of conserved meiosis related genes.
